# Category-like representation of statistical regularities allows for stable distractor suppression

**DOI:** 10.3389/fpsyg.2025.1598594

**Published:** 2025-08-06

**Authors:** Catherine W. Seitz, Anthony W. Sali

**Affiliations:** Department of Psychology, Wake Forest University, Winston-Salem, NC, United States

**Keywords:** attentional capture, distractor suppression, statistical learning, computational modeling, attention mechanism

## Abstract

Statistical learning allows observers to suppress attentional capture by salient singleton distractors appearing at a predictable location. This form of learning is in many cases inflexible, persisting into extinction and across changes in probability without strong contextual cues. However, the underlying learning mechanisms and nature of distractor probability representations remain unclear. Here, we replicated learned location-based distractor suppression in two experiments, both showing a reduction in attentional capture when a salient distractor appeared at a high-probability distractor location, alongside impairments in target selection at that same location. We then used computational modeling to explore whether the strength of suppression was best explained by continuous distractor frequency summation, reinforcement learning prediction errors, or a categorical all-or-nothing response. In both experiments, our data were most parsimoniously explained by a combination of a global exponential decay in response time with each distractor presentation paired with a categorical learning mechanism in which the most highly associated location was suppressed over all other locations. Our results suggest that the magnitude of suppression is more sensitive to overall probability differences than to subtle trial histories.

## Introduction

The likelihood that attention will select an object or spatial location, referred to as attentional priority, varies in part as a function of our previous experiences (Awh et al., 2012; Anderson et al., 2021). These selection history effects span both short time scales, such as the facilitation or inhibition of target and distractor stimuli following the priming of features and locations (Maljkovic and Nakayama, 1994, 1996), and longer time scales, such as the involuntary orienting of attention toward stimuli that are associated with reward (Anderson et al., 2011). Using the statistical regularities present in the environment, individuals learn to anticipate the spatial location of targets, facilitating visual search (Chun and Jiang, 1998; Geng and Behrmann, 2002, 2005). More recent evidence has shown that statistical structure guides the suppression of attentional selection, allowing individuals to inhibit the tendency to attend to salient, but task-irrelevant, distractors (Gaspelin and Luck, 2019). One such form of distractor suppression results from the association of spatial locations with a high probability of containing a salient distractor (Wang and Theeuwes, 2018a, 2018b, 2018c). However, the processes regulating learned distractor suppression remain unknown. The visual system must at times flexibly update distractor predictions in response to underlying changes in the environment yet at other times maintain stable representations of priority even in the face of periodic unexpected outcomes. In the current study, we employ a series of computational models to test how individuals track location-based distractor likelihoods and use these representations to avoid attentional capture.

A growing body of work has described the ways in which location-based statistical regularities influence the suppression of task-irrelevant distractors. Individuals inhibit the selection of an additional singleton distractor when it frequently appears at a predictable spatial location, termed the high probability location, relative to other potential locations, termed low probability locations (Wang and Theeuwes, 2018a, 2018b). Likewise, targets appearing at this high probability location on distractor absent trials are identified more slowly than those appearing at low probability locations. Learned location-based distractor suppression reflects a preparatory process such that participants are slower to detect a probe dot appearing at a high probability location than at low probability locations even before the search display onset (Huang et al., 2023). Location-defined regularities bias attentional priority in both singleton detection and feature search modes (Wang and Theeuwes, 2018c), produce varying magnitudes of suppression that increase as salience increases, (Failing and Theeuwes, 2020), and lead to suppression that cannot be accounted for by statistical regularities governing the appearance of the target (Failing et al., 2019).

Understanding the flexibility of statistical learning is important for characterizing the degree to which the visual system can reconfigure in response to changes in the environment. Some evidence suggests that this learning is relatively inflexible. Individuals continue to suppress old high probability distractor locations into extinction phases and learned suppression extends across cued contexts (Britton and Anderson, 2020; de Waard et al., 2022). Conversely, in a more recent experiment in which task type (compound search vs. detection) served as a context cue and participants were given extended training on each task, participants showed evidence of context-dependent learning (de Waard et al., 2023). Participants suppressed context-specific high probability locations when distractor location probabilities were biased and experienced a reinstatement of suppression for a previously learned context-dependent high probability location when reentering a previous context with unbiased location probabilities. However, in this reinstatement test phase, they also demonstrated equal suppression of other previous high probability locations, regardless of the current context cue. Taken together, statistically learned distractor suppression can be context-specific, but in the absence of new biased regularities, old learning persists, remaining relatively inflexible and resilient to extinction.

Despite our growing understanding of the ways in which statistical learning informs location-based distractor suppression, the nature of distractor probability representations and the mechanism used to update these representations are unknown. The suppression effect linearly scales with the ratio of high to low distractor location probabilities, suggesting that individuals represent the differences in likelihood (Lin et al., 2021). Consequently, one possibility is that the magnitude of suppression afforded to a particular location is proportional to the individual’s estimate of that location-specific distractor likelihood. Conversely, it is possible that trial-by-trial suppression is governed by a rule-like representation of high and low probability locations, shielding settings of attentional priority from occasional unexpected outcomes and applying suppression to a privileged location or locations in an all-or-nothing fashion. The instantiation of location-based learned suppression does not readily decrease after the removal of biased probabilities (Britton and Anderson, 2020). Participants may therefore quickly settle on a categorical rule-like representation of the to-be-suppressed locations that is insensitive to subtle differences in distractor history among unprivileged locations.

If distractor likelihood representations are continuous in nature, the mechanism responsible for updating representations may carry a greater significance in shaping the impact on settings of attentional priority. One potential updating approach is the independent summation of distractor occurrences for each location. Under this model, an increase in the estimated likelihood of a distractor appearing at one location does not decrease the estimate for another location. Thus, while the relative strength of suppression at a location may decrease as suppression at other locations increases, the raw magnitude only increases or remains the same. Here, we refer to this potential mechanism as the accumulator hypothesis.

Alternatively, reinforcement learning (RL) also offers a potential explanation for how individuals integrate information about distractor location probabilities over time. RL models stipulate that individuals update predictions in response to violations of expectations, referred to as prediction errors (Sutton and Barto, 1998). Even in the absence of explicit rewards, correct performance is intrinsically rewarding, and individuals are motivated to accurately find targets in visual search tasks. RL has been implicated in a variety of areas of cognitive control that are similarly impacted by statistical structure such as conflict control (Chiu and Egner, 2019), task-switching flexibility (Sali et al., 2024), and attention shifting readiness (Sali et al., 2020; for a review, see Braem and Egner, 2018). Therefore, it is possible that individuals maintain and update predictions about where a distractor will appear dynamically based on the magnitude of the difference between their current predictions of distractor likelihoods and the outcome of a trial, known as the prediction error (PE).

In addition to stable settings of attentional priority that are based on long trial histories, there is also considerable evidence that short-term priming plays a significant role in the setting of attentional priority. Visual search is facilitated when stimulus features, such as color or location, repeat (Maljkovic and Nakayama, 1994, 1996). These effects span 6–8 trials for both color and location priming and are cumulative such that repeated instances of a particular feature or location lead to increasing facilitation. Given the usual manipulation of distractor frequencies, a trial in which a distractor appears at a high probability location is more likely to follow another high probability distractor trial than a trial with a low probability distractor. However, several studies have demonstrated that when manipulating distractor location frequencies, RTs for high probability trials following other high probability trials do not significantly differ from those following low probability trials (Wang and Theeuwes, 2018b, 2018a). Furthermore, learned suppression would not persist into extinction phases if it were dependent on trial-by-trial priming alone (Britton and Anderson, 2020). While it seems unlikely that priming is the sole contributor to location-based distractor suppression, moment-by-moment changes in priority may reflect a combination of priming and statistical learning, with priming serving as a potential mechanism that drives performance during initial learning (Anderson et al., 2021).

In the current study, we examined how individuals track distractor location probabilities to set attentional priority. Participants searched for a shape singleton target among homogenous non-target stimuli. On some trials, one of the non-target stimuli, referred to as the distractor, appeared in a different color than the other items in the search array. As in previous studies, we manipulated the likelihood of a salient color singleton distractor appearing at each location in the search array across individuals. Thus, there was always a location that was associated with a high probability of a distractor appearing with the remaining locations having a low probability of containing a distractor. In Experiment 1, each participant received a constant set of probabilities such that the high probability location never changed. In Experiment 2, half of the participants received constant probabilities (as in the previous experiment), and the other half of participants periodically experienced a change in the high probability distractor location. Since our focus here was on the trial-by-trial adjustment in distractor probability estimation rather than context-based learning, we did not vary context cues as in some previous studies (Britton and Anderson, 2020; de Waard et al., 2023).

To examine the nature of distractor location predictions, we fit behavioral RT data from trials with a salient color distractor with a series of computational models that corresponded to the accumulator, RL, and categorical hypotheses. In each case, we included an additional regressor in the model that accounted for global changes in capture not arising from learned statistical regularities and a regressor that accounted for whether the previous trial contained a distractor. First, we sought to determine which hypothesis most parsimoniously accounted for our results. Second, we conducted model recovery simulations in which we tested the extent to which data generated by our models were accurately recovered by an identical model comparison procedure. Importantly, this analysis addresses a slightly different question than the model comparison by testing whether accumulator, RL, and categorical mechanisms would make similar predictions about performance given the best-fit model parameters derived from our data and the remaining unexplained variability in RTs after model fitting.

For each hypothesis, we tested models that tracked distractor probabilities across blocks of the task as well as models that reset predictions with each block. In a stable environment in which probabilities are unchanging (Experiment 1), maintaining predictions across blocks minimizes noise in predictions at the start of each block and maintains a stable representation of distractor likelihood. However, periodic resetting of predictions affords greater flexibility and may be advantageous when distractor likelihoods periodically change (Experiment 2). In addition to the models described above, we also included models that accounted for evidence accumulation without a global trend in the magnitude of capture.

## Experiment 1

In Experiment 1, we manipulated distractor likelihoods across spatial locations with statistical regularities that remained constant. The degree to which individuals weight recent experiences above long-term trial histories, referred to as the temporal integration window, is central to the current study. For example, a distractor appearing at an unexpected location could lead to rapid adjustment in prioritization for upcoming trials or could play a minor role in future priority adjustments as one small contradictory piece of evidence within a longer trial history.

With a long temporal integration window, the accumulator and RL hypotheses make similar predictions about statistical learning that could appear categorical in nature when the behavioral signatures generated by the learning mechanism are smaller than unexplained trial-by-trial noise in RT. Under the accumulator hypothesis, the magnitude of suppression at a particular location reflects the total number of times a distractor has previously appeared at that location. If each presentation of a distractor is weighted equally, rare low probability distractor presentations will have little impact in the overall setting of priority. Likewise, if prediction updates arising from PE are small, rare distractor occurrences will do little to change the overall predictions. These category-like representations of high and low probability distractor locations would be sensitive to probability differences across locations but not noticeably vary in strength over time based on the number of previous distractor occurrences at a given location. Support for the categorical hypothesis is therefore not at odds with either the accumulator or RL hypotheses as it offers a more parsimonious explanation of a potentially similar behavioral profile. Rather, it would still be possible that the behavioral signatures of more nuanced distractor likelihood representations are too small to reliably detect given other sources of RT variability.

The predictions made by the accumulator and RL accounts diverge as the temporal integration window decreases. In contrast to the gradual accumulation of evidence described above, the initial decrease in priority in response to early distractor presentations at a particular location may be greater than those after later presentations, reflecting an exponential decline in the magnitude of location-based suppression. Furthermore, the accumulator hypothesis predicts that suppression is never reduced for a given location and differences in the magnitude of suppression across locations will decrease over time as low probability locations receive greater suppression. Conversely, short temporal integration under RL would be signified by individuals suppressing locations that have recently contained a distractor above those that have a long-term history of distractor association.

Taken together, evidence in favor of category-like suppression, a low-decay accumulator, or a low learning rate RL mechanism would favor a stable statistical learning mechanism that integrates trial histories broadly, while support for higher decay accumulator or higher learning rate RL mechanisms would shed light on the ways in which distractor suppression learning flexibly integrates new information into ongoing representations.

### Materials and methods

#### Participants

Twenty-one participants (12 female, ages: *M* = 18.8, *SD* = 1.1) were recruited through Wake Forest University for credit in an undergraduate Introductory Psychology course. We chose this sample size based on previous studies with similar sizes (Wang and Theeuwes, 2018a). Four participants were excluded from the final data analysis due to a bug in the stimulus presentation code that incorrectly set distractor location probabilities. A power analysis in G*Power based on the effect size of Wang and Theeuwes’s (2018a) test of RT differences across distractor locations (Experiment 1; *η_p_^2^* = 0.74) revealed that we would need to run six participants to reach 95% power. We used Hierarchical Bayesian Inference (HBI) for model fitting and comparison (Piray et al., 2019). Previous simulations have demonstrated that HBI model comparison performs well across scenarios with different numbers of participants, trials, and parameters. Here, we rely on the protected exceedance probability (pxp) for model comparison. This value reflects the probability that a given model best explains the data across the sample, accounting for the possibility that there may be no difference between models. In a previous study, even when requiring the protected exceedance probability to be greater than 0.95 for model selection, HBI selected the correct model on over 80% of simulations with a sample size of 16 participants (Piray et al., 2019). All participants had normal or corrected-to-normal color vision. The study was approved by the Wake Forest University Institutional Review Board and all participants provided written informed consent.

#### Apparatus

Participants completed the experiment in a dimly lit room with a Samsung S24C200 monitor positioned at a viewing distance of approximately 66 cm. Participants made all responses using a standard computer keyboard. Stimulus presentation was controlled by the Psychophysics Toolbox running in MATLAB (Brainard, 1997). We did not control head position with a chin rest, and consequently, all visual angle measurements reported below are approximate.

#### Design and procedure

**Figure 1:**
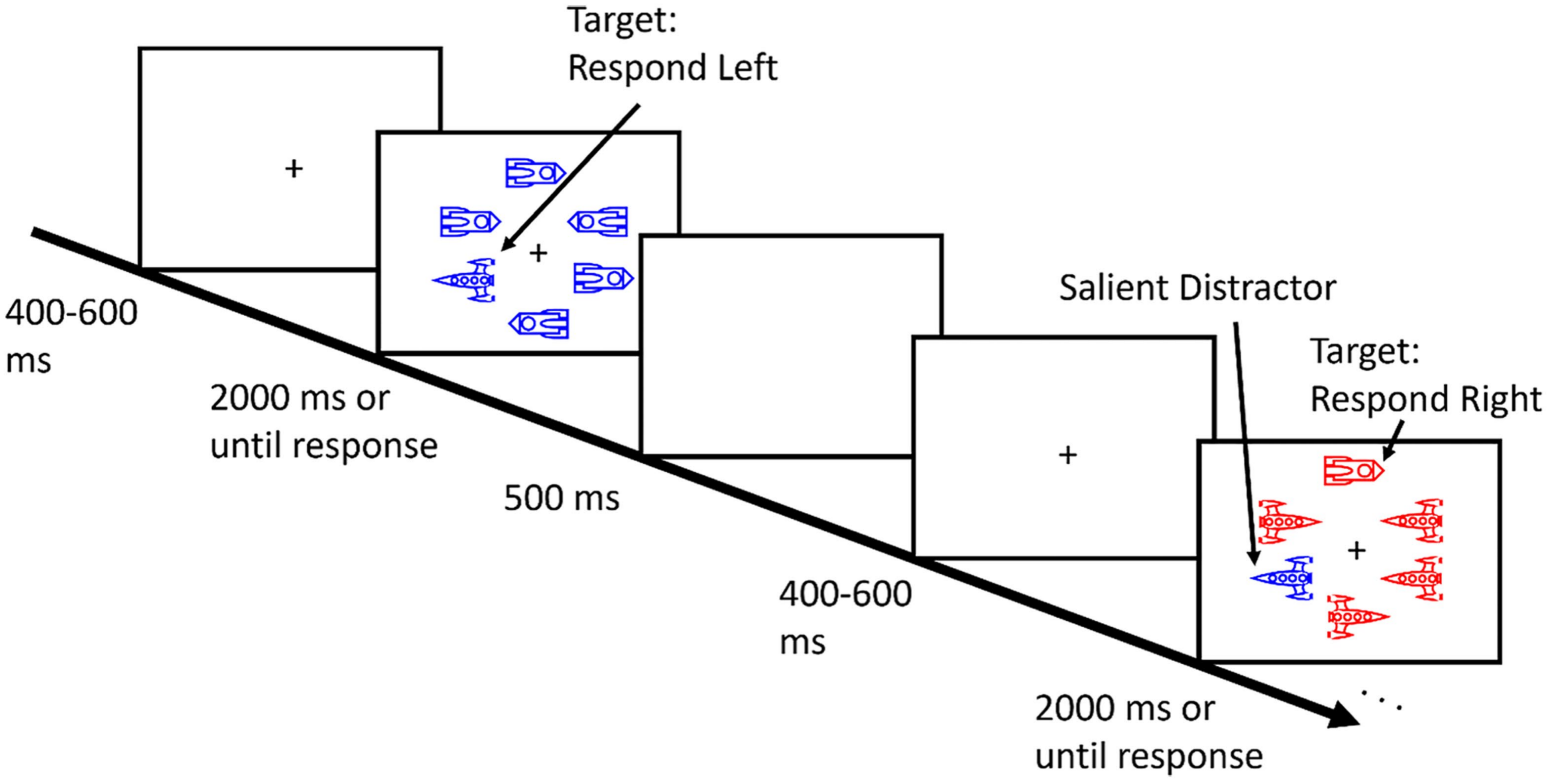
Example trials of the additional singleton search task with spaceships as search items.

Participants completed a variant of the additional singleton paradigm used by Wang and Theeuwes (2018a). Each trial began with a white cross presented in the center of a black display for 400, 500, or 600 ms. Following this variable interval, a search display of 6 items appeared in an imaginary circle around the fixation cross (1° × 1°) with a radius of 7° of visual angle. Each item subtended approximately 3° × 2°. As illustrated in Figure 1, the items in the visual search array were spaceships and each array consisted of two spaceship shapes such that there were always 5 of one type and 1 of the other (Gaspelin et al., 2015). Participants searched for the unique spaceship shape and indicated the direction it was facing by pressing the “Z” key if this target faced left and the “M” key if it faced right. Each array was presented until the response or for a maximum of 2000 ms. If a key was not pressed within that timeframe, the next trial began and that trial was marked as incorrect. Participants completed 480 trials in total, broken up into 8 blocks of 60 trials each. In between each block, participants took a brief break and received accuracy feedback. Participants completed a series of 18 practice trials prior to beginning the main task. Following the visual search task, participants also completed a visual change detection working memory task (data not reported here).

The stimuli appeared in either red (RGB: 255,0,0) or blue (RGB: 0,0,255) and on 50% of trials, a salient color singleton appeared at one of the non-target locations (i.e., a red spaceship among 5 blue spaceships or vice versa). The colors of the stimuli were randomized across trials. Importantly, this meant that while trial-by-trial changes in color might influence the magnitude of attentional capture, color priming could not account for any of the results reported here. As in previous tests of spatial learned distractor suppression, (e.g., Wang and Theeuwes, 2018b, 2018a, 2018c), we manipulated the frequency with which this color singleton appeared at each of the 6 potential locations. On 66.67% of distractor present trials, the color-singleton appeared at a particular location in the array (counterbalanced across participants, with the exception of one location due to the participant exclusion described above), termed the high-probability location. On the rest of the distractor present trials, the distractor appeared equally at the other 5 locations.

The target appeared at each location in the array the same number of times per block so that the likelihood of target repetitions across high probability, low probability, and absent conditions was equated. For distractor absent trials, target position was balanced across locations. Since the target could not appear at the high probability location for high probability distractor trials, half of all low probability distractor trials had the target appear at the high probability location in compensation, balancing target position for distractor present trials. The target position on the remaining low probability distractor trials was balanced among the low probability locations with the position determined according to a set of predefined distractor-target location relationships that repeated across blocks and participants (see Experiment 2 for a design that randomizes the distractor-target relationship for these trials where the target did not appear at the high probability location). Although this dependence meant that distractor position predicted target location on low probability distractor trials, such an effect would only speed performance and lower the RT difference between high probability and low probability distractor location trials. Thus, evidence of increased attentional capture on low probability trials would be consistent with an explanation of distractor likelihood statistical learning. Due to a technical error, there was an imbalance in the target locations for high probability distractor trials for three participants such that one low probability location received an extra target per run and another location received one fewer target per run. Although the likelihood of the target appearing at one location was slightly increased, this was offset by a corresponding decrease in the likelihood of target appearances at another location, leaving the overall balance of target presentations at the high and low probability distractor locations equated.

#### Data processing

Inaccurate trials and trials with RTs less than 200 milliseconds (ms) were removed from all RT analyses. We adopted the fixed cutoff of 200 ms to avoid biases from adaptive procedures (Miller, 2023). This amounted to less than 1% of accurate response trials being removed from RT analyses. The response deadline of 2000 ms limited the extent to which trials with long RTs could impact the analysis. All trials were included in the accuracy analyses, regardless of RT.

#### Computational modeling

We used a series of computational models to test the underlying mechanisms of learned distractor suppression. In all cases, we predicted trial-by-trial RTs for distractor present trials only since those are the trials for which we could measure distractor suppression. We excluded the first trial of each block because this trial could not be categorized based on previous distractor presence, trials without an accurate response, and trials with an RT flagged as an outlier (see above). Furthermore, we *z*-transformed all continuous regressors. In each case, we predicted trial-by-trial RTs with a general linear model (GLM). We estimated all parameters using HBI from the CBM toolbox (Piray et al., 2019). This procedure regularizes parameters for each participant according to the degree to which each participant’s data can be accounted for by a given model, termed responsibility, while also simultaneously providing a random effects comparison of models to estimate the probability that one model best explains the group data. The HBI procedure operates iteratively, setting each model’s group parameters with a greater weighting of results derived from participants with high responsibility. Given this, even models with weak initial advantages may eventually come to best capture data across the entire sample and it is important to keep in mind that the pxp values reported here reflect a model comparison at the conclusion of this iterative process.

To fit each model, HBI maximizes the log likelihood, which was computed as $\mathrm{LL}=\sum (-0.5*{\left(\frac{y_{i}-\mu_{i}}{\hat{\sigma }}\right)}^{2}-\ln(\sqrt{2\pi }*\hat{\sigma }))$ , where *y*_i_ was the recorded RT on trial *i* in units of seconds and $\mu_{i}$ was the predicted RT for trial *i,* using a nonlinear optimization procedure. Modeling RTs in units of seconds instead of milliseconds allowed us to use the same prior variance for all parameters (see below). At each iteration of parameter optimization for each participant, we re-estimated the standard deviation of errors ( $\hat{\sigma }$ ) according to: $\hat{\sigma }=\sqrt{\frac{\mathrm{RSS}}{n}}$ , where *n* denotes the number of data points included in the GLM and *RSS* denotes the residual sum of squared errors, to compute the log likelihood. Each parameter was given a prior with a mean of 0 and a variance of 6.25 as in previous studies (Piray et al., 2014, 2019). We used all defaults from the CBM toolbox, except setting the objective limit of the nonlinear optimization to −10^20^, as is the default in MATLAB.

Unless noted below, each model contained an exponential decay regressor that accounted for global changes in distractor suppression across all distractor present trials and a dummy-coded regressor that coded whether the previous trial had a color singleton distractor present (1 = distractor absent, 0 = distractor present). We elected to omit an additional regressor that accounted for distractor location repetitions since this labeling was not orthogonal to our learning mechanisms of interest given the greater number of high probability distractor trials than low probability distractor trials (but see Model 7, which takes this approach instead of a learning mechanism). The decay regressor was defined as $\text{GlobalDeca}y_{i+1}=e^{-b_{\text{global}}*i}$ , where *b_global_* was a free parameter representing the rate of decay and *i* was the distractor present trial number. For the first trial (*i* = 1), global decay was equal to 1. Since HBI assumes that all parameters are normally distributed around some mean, we constrained the decay rate to positive values only with an exponential transformation. Below, we describe each model in more detail.

Model 1 explained performance according to the frequency of distractor appearances at each location. To account for the possibility that the rate at which distractor presentations decrease priority may decelerate over time, we updated distractor associations for each of the six potential locations (*pDist_l,i_*) according to separate exponential decay functions. First, we computed the frequency of distractor presentations at each location as $c_{l,i+1}=c_{l,i}+\mathrm{Dis}t_{l,i}$ , where *l* indicates the search array location, *i* indicates the trial number, and *Dist* was either a 1 if a distractor was present or a 0 if absent at the corresponding location. After these updates, we applied the exponential decay function as $\text{pDis}t_{l,i+1}=1-e^{-b_{\text{distractor}}*c_{l,i+1}}$ , where *b_distractor_* was the decay rate for location-based updating. The location-based updating decay rate was constrained to be positive with an exponential transformation. After updating distractor predictions across all trials, we formed a GLM regressor according to these location-specific distractor predictions by selecting the prediction associated with the location of the distractor for each trial. In addition to this distractor prediction regressor, we also included regressors that accounted for global decay in RT, whether the preceding trial had a distractor present (see above), as well as an intercept. Therefore, Model 1 contained a total of 6 free parameters to fit with HBI.

Model 2 used RL to update distractor predictions for each location according to the magnitude of distractor PE, defined as the difference between the distractor outcome for the location of interest (*Dist*; coded as 1 = present; 0 = absent) and the current model-based distractor prediction for this location (*pDist_l,i_*). Each location (*l*) was updated according to: $\text{pDis}t_{l,i+1}=\text{pDis}t_{l,i}+\alpha *({\text{Dist}}_{l,i}-\text{pDis}t_{l,i}),$ where α denotes the learning rate, which controls whether predictions vary based on immediate trial history or integrate evidence across longer timescales (Rescorla and Wagner, 1972). We applied a sigmoid transform to constrain α to fall between 0 and 1. As in the other models, distractor predictions were updated according to distractor present trials only, meaning that predictions did not decrease on trials in which there was no distractor present. We initialized the distractor prediction for each location at 0.16667. For the GLM, we constructed the distractor prediction regressor by selecting the prediction (*pDist_l,i_*) associated with the current trial’s distractor location. As in Model 1, there were a total of 6 free parameters.

Model 3 updated frequency accounts as in Model 1 but differed in how the distractor predictions were used to predict trial-by-trial RT. Model 1 allowed for a graded representation of suppression such that the strength of suppression was dependent on trial history. Conversely, Model 3 accounted for the possibility that suppression operates in a categorical all-or-nothing fashion, with maximum suppression always applied to the location most highly associated with a future distractor. All aspects of Model 3 were identical to Model 1, except instead of applying an exponential decay function to the distractor frequencies, we assigned a regressor a 1 if the distractor was at the current maximum distractor prediction location and a 0 otherwise. We allowed ties when computing the maximum distractor prediction location such that the regressor was given a 1 if the current distractor location was one of the tied locations. Thus, unlike Model 1, this model applied suppression in a dichotomous fashion, resulting in a total of 5 free parameters. While our test of the categorical hypothesis was paired with a probability tracking mechanism that relies on summation alone, we revisit in the discussion how categorical suppression could be paired with other forms of learning.

Each of the models discussed so far carried previous distractor predictions across blocks. To account for the possibility that some participants may reset expectations at the start of each block, we included a set of 3 additional models. Models 4 and 6 were identical to Models 1 and 3, respectively, but reset all frequencies to 0 at the start of each block. Model 5 was identical to Model 2 but reset model-derived distractor predictions to 0.16667 at the start of each block.

Model 7 accounted for RT according to only global RT decay and immediate trial history. In addition to a regressor that coded whether the previous trial had a distractor present, we included another dummy coded regressor that indicated whether the distractor location was the same as the previous trial (distractor repetition = 1, distractor change = 0). The combination of dummy-coded regressors therefore represented three potential trial types (previous trial distractor absent, distractor location repetition, distractor location change), yielding 5 free parameters. Unlike the other models, Model 7 represented only the impact of trial-by-trial priming without any longer-term statistical learning.

Finally, Models 8 and 9 were identical to Models 1 and 4, respectively, but did not include the global decay regressor. We included these models to determine whether the location-specific decay functions would together best account for overall trends in RT across the experiment without the need of a global regressor.

#### Model recovery simulations

We conducted a series of simulations to determine the extent to which our model comparison procedure could reliably identify data generated by the accumulator, RL, and categorical hypotheses given the obtained best-fit parameters from our observed data. We reran HBI for each model in isolation to obtain estimates of the group mean for each parameter and of the variability associated with each parameter across participants, referred to as the hierarchical error. After obtaining the regularized best-fit parameters for each participant and for each model, we computed the standard deviation of each residual RT time series to estimate the variability in our data not explained by each of the models.

We conducted simulations under conditions of low and high noise. Due to the computational demands of these simulations, we elected to generate data from just Models 1, 2, 3, and 7 to determine the extent to which data generated from the accumulator, RL, and category hypotheses without resetting could mimic each other or our model with trial-by-trial priming. We generated 30 sets of parameters for each model by drawing from normal distributions with the mean set to the parameter’s group mean from HBI and the standard deviation set to the group hierarchical error. We then used these parameters to simulate RTs for distractor present trials based on the actual trial sequences of the 17 participants included in our main analyses. So that our simulation would match our observed data as closely as possible, we applied the trial-by-trial labels of accurate and inaccurate from our observed data to the simulated datasets, yielding simulations with the same number of trials as in our observed dataset. For each trial, we added noise that was randomly drawn from a normal distribution. We first fixed the standard deviation of the noise distribution to 50 ms for each participant to test model recovery under conditions of low noise. Next, we repeated the simulation under conditions of noise that matched those in our observed data, using the same underlying model-generated data and random number seeds. We randomly assigned one of the actual residual standard deviations from our observed data fits to a simulated participant for each iteration, sampling without replacement, and using the assigned standard deviation to again randomly inject noise into the simulated data. In both simulations, we clipped any RTs falling above 2000 ms or below 200 ms to preserve the range of RTs included in the original analysis. We then ran HBI on the model-generated datasets, separately for conditions of low and high noise, and selected the winning model in each case as the model with the highest exceedance probability. The maximum number of HBI iterations for each simulation was set to 50.

#### Data analysis

Both experiments were approved by the Wake Forest University Institutional Review Board and data collection took place between 2022 and 2024. Analyses were conducted in MATLAB R2024a (MATLAB version 24.1.0.2578822 (r2024a) update 2, 2024) and R version 4.2 (R Core Team, 2024) with the following packages: *tidyverse* version 2.0.0 (Wickham et al., 2019), *tidyr* version 1.3.1 (Wickham et al., 2024), *rstatix* version 0.7.2 (Kassambara, 2023), *data.table* version 1.16.4 (Barrett et al., 2024), *afex* version 1.4-1 (Singmann et al., 2024)*, cowplot* version 1.1.3 (Wilke, 2024)*, superb* version 0.95.15 (Cousineau et al., 2021), and *R.matlab* version 3.7.0 (Bengtsson, 2022). Neither experiment’s design nor analyses were pre-registered.

#### Data availability

All data, stimulus presentation code, and analysis code are available at https://osf.io/j7bn2/.

### Results

**Figure 2:**
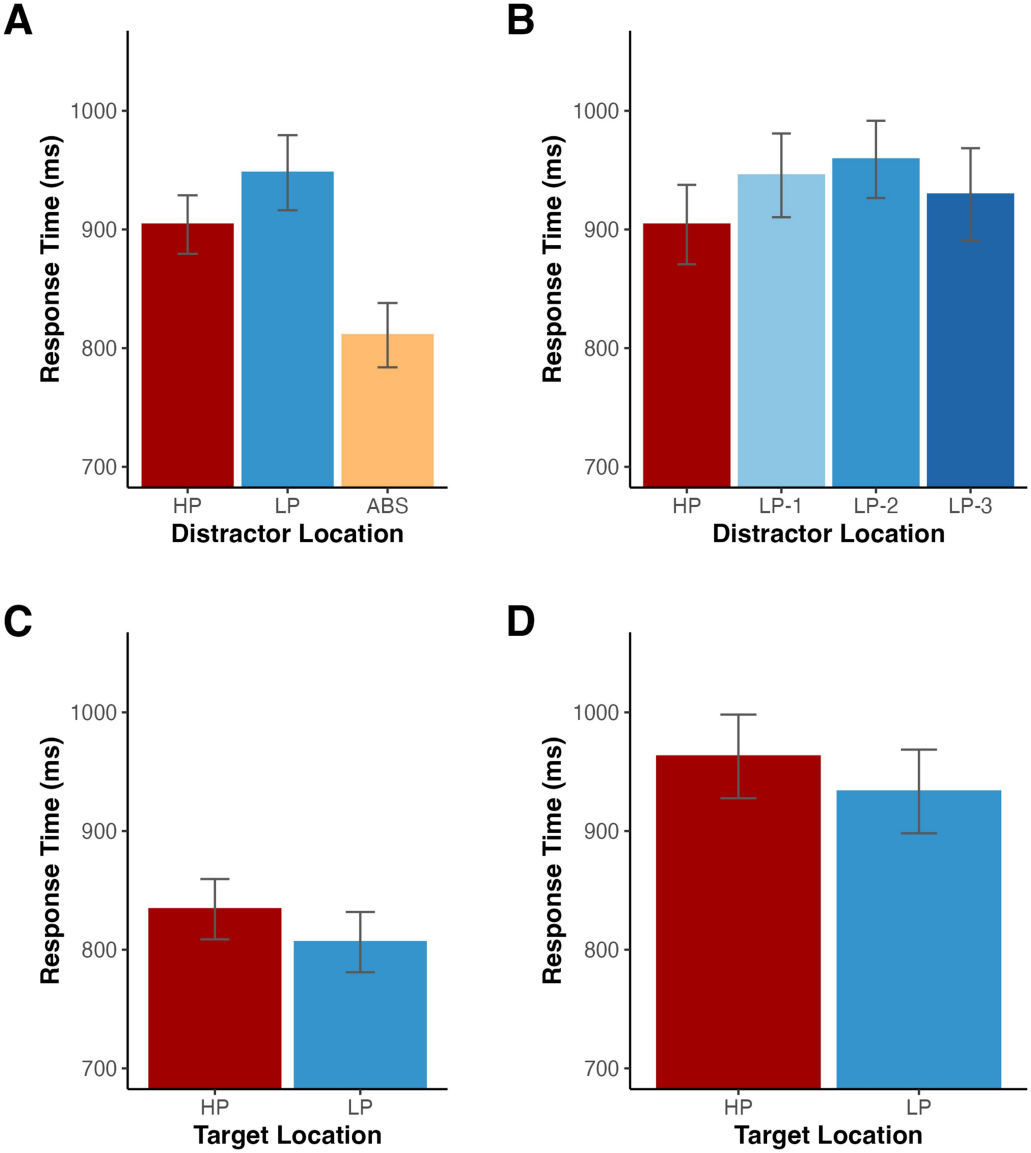
**(A,B)** Mean RTs depending on the distractor location for Experiment 1. **(C)** Mean RTs for distractor absent trials depending on the target location for Experiment 1. **(D)** Mean RTs for low probability distractor trials depending on the target location for Experiment 1. HP indicates the high-probability location, LP-1 indicates one item away from the high-probability location, LP-2 indicates two items away, LP-3 indicates three items away, and ABS indicates when the distractor was absent. Error bars denote difference- and correlation-adjusted 95% confidence intervals (Cousineau et al., 2021). Correlation adjustment was carried out using the Cousineau-Morey approach (Cousineau, 2005; Morey, 2008).

Before turning to the computational modeling, we first conducted more traditional RT and accuracy analyses to determine whether we observed evidence of distractor suppression. A repeated measures ANOVA on mean RTs with distractor location (high-probability, low-probability, and distractor absent) as a factor revealed a significant difference among distractor locations, *F*(2, 32) = 56.27, *p* < 0.001, *η_p_^2^* = 0.779 (Figure 2A). To better characterize this significant effect, we conducted a series of post-hoc comparisons with Holm-Bonferroni correction for multiple comparisons. Relative to distractor absent trials, participants were significantly slower when the distractor was present in the low-probability location, *t*(16) = 9.40, *p* < 0.001, *d*_z_ = 2.28, and when the distractor was present in the high-probability location, *t*(16) = 8.34, *p* < 0.001, *d*_z_ = 2.02. Importantly, RTs were also faster when the distractor appeared in the high-probability location compared to the low-probability locations, *t*(16) = 3.22, *p* = 0.005, *d*_z_ = 0.78, suggesting individuals learned to suppress the high probability distractor location.

Since some studies (Wang and Theeuwes, 2018b) have reported a spatial gradient of suppression, such that individuals gradually suppress locations nearby the high-probability location, we split the low-probability condition into three trial types based on the distance from the high-probability location. Using an ANOVA on RTs with distractor location (high-probability, one item away from high-probability location, two items away from high-probability location, and three items away from high-probability location) as a factor, we found a significant difference among distractor locations, *F*(3,48) = 4.06, *p* = 0.012, *η_p_^2^* = 0.202 (Figure 2B). To further examine the relationship among RTs for all locations, we fit each participant’s RT averages for each low probability distractor location with a linear model and tested whether the slopes significantly differed from zero across participants. On average, there was no significant slope, *t*(16) = −0.83, *p* = 0.420, d_z_ = −0.20, suggesting that while participants were slower when the distractor appeared at the low probability location relative to the high probability location, there was not a significant gradient of suppression (*M* = −8.06 ms, *SD* = 40.18).

We also examined the effect on target selection when the target appeared in the high-probability and low-probability distractor locations. First, we tested distractor absent trials. RTs were slower when the target appeared in the high-probability location compared to the low-probability locations, *t*(16) = 2.32, *p* = 0.034, *d*_z_ = 0.56, providing further evidence for location-specific suppression (see Figure 2C). Additionally, targets appeared at high probability distractor locations on half of all low probability distractor trials, creating an imbalance in the number of low probability distractor trials with a target in each position. When testing these trials, there was a trend such that participants had slower RTs when the target appeared at the high probability location, *t*(16) = 1.78, *p* = 0.095, *d*_z_ = 0.43 (see Figure 2D).

We conducted a parallel analysis of behavioral accuracies for absent, low distractor probability and high distractor probability trials, *F*(2,32) = 13.34, *p* < 0.001 (Geisser–Greenhouse corrected for violation of the sphericity assumption), *η_p_^2^* = 0.455 (see Table 1). As above, we used a Holm-Bonferroni correction and found significantly lower accuracies when the distractor appeared in a low-probability location, *t*(16) = 3.89, *p* = 0.003, *d*_z_ = 0.94, and when the distractor appeared in the high-probability location, *t*(16) = 5.03, *p* < 0.001, *d*_z_ = 1.22, relative to the distractor absent condition. There was a trend such that accuracies were higher when the distractor appeared in the high-probability location compared to the low-probability locations, *t*(16) = 2.00, *p* = 0.062, *d*_z_ = 0.49.

**Table 1:** Behavioral accuracies for Experiment 1.

High probability distractor	Low probability distractor	Distractor absent
95.81 (2.36)	94.71 (3.89)	97.72 (1.65)

Mean accuracy (SD).

#### Mechanisms of learning

**Figure 3:**
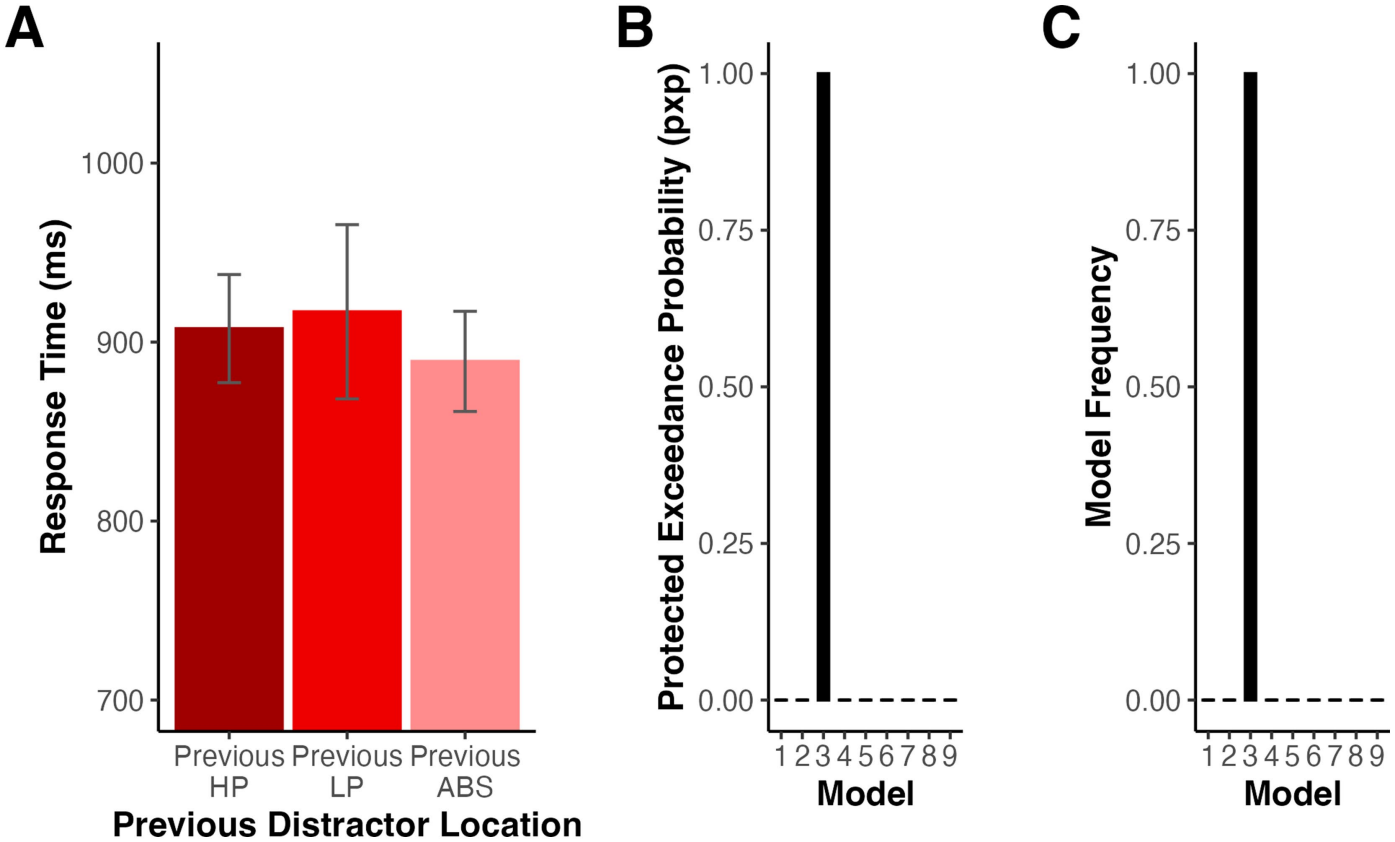
**(A)** Mean RTs for high probability distractor trials depending on the previous trial type for Experiment 1. Error bars denote difference- and correlation-adjusted 95% confidence intervals (Cousineau et al., 2021). Correlation adjustment was carried out using the Cousineau-Morey approach (Cousineau, 2005; Morey, 2008). **(B)** Protected Exceedance Probability for model comparison. **(C)** Proportion of participants whose data were best explained by each model.

We first examined the impact of immediate trial history on distractor present trial RTs. As in previous studies, we tested whether RTs on high probability distractor present trials differed for trials following a high probability distractor relative to those following a low probability distractor to determine whether our results could be accounted for by distractor location repetition priming. For this analysis and all subsequent modeling, we excluded the first trial of each block. In this analysis, we also excluded trials where the target location matched the preceding trial’s target location to isolate the effect of previous distractor condition because target repetitions differed in likelihood for high probability trials following each trial type given our overall probability manipulation (see Method). A repeated measures ANOVA revealed that there were no significant differences in RT between high probability distractor trials following high probability distractor, low probability distractor, and absent trials, *F*(2,32) = 1.32, *p* = 0.275 (Geisser–Greenhouse corrected for violation of the sphericity assumption), *η_p_^2^* = 0.076 (see Figure 3A).

We next used HBI to simultaneously fit and compare a series of models (see Method) to determine how short- and long-term trial histories influenced the moment-by-moment instantiation of learned distractor suppression. This procedure regularized parameter fits according to the extent to which each model best explained a given participant’s data. We tested whether trial-by-trial changes in RT were best explained by graded suppression according to frequency accumulation (Models 1 and 4), model-derived distractor PE (Models 2 and 5), categorical suppression of the strongest associated location (Models 3 and 6), or only immediate trial history (Model 7). Two additional models tested frequency accumulation without the global trend regressor (Models 8 and 9). In all cases, we fit the data for distractor present trials and used the pxp to identify which model was able to best explain the data across our sample.

As illustrated in Figure 3B, the data were best explained by a combination of global exponential decline and the dichotomous categorization of high vs. low likelihood distractor locations (Model 3) with a pxp of 1.00 and a Bayesian omnibus risk (bor), the likelihood that there is no significant difference in the likelihood of the tested models, less than 0.001. Figure 3C depicts the model frequencies, the proportion of participants whose data were best explained by each model at the conclusion of HBI. To assess Model 3’s fit, we reran single participant GLMs with the best-fit individual parameters derived from the HBI procedure. The resulting *R^2^* values ranged from 0.05–0.33 (*M* = 0.17, *SD* = 0.07).

For the winning model, we further investigated the best-fit group parameters, which weight the individual participants’ data according to the degree to which the model was deemed responsible for that participant’s performance (see Piray et al., 2019). The HBI-derived group parameters and estimated hierarchical error are shown in Table 2. Given the model fitting procedure, the estimated parameters for each participant are not independent, thus violating an assumption of traditional parametric statistics. We therefore used Piray et al.’s (2019) HBI *t*-test to determine whether the group GLM parameter estimates for Model 3 significantly differed from 0. As in the ANOVA, a significant negative relationship between the distractor prediction regressor and RT indicated that participants demonstrated larger attentional capture on low probability distractor trials than on high probability distractor trials *t_HBI_*(18.00) = −3.01, *p* = 0.008. The exponential decline regressor also significantly accounted for the trial-by-trial RTs *t_HBI_*(18.00) = 7.48, *p* < 0.001. However, there was no significant change in RT based on whether the previous trial had a distractor present, *t_HBI_*(18.00) = −0.55, *p* = 0.591.

**Table 2:** Group parameters for Model 3 in Experiment 1.

Distractor prediction parameter estimate	Global decay parameter estimate	Decay rate	Previous trial distractor absent parameter estimate	Intercept
−0.040 (−0.053 – −0.027)	0.092 (0.080 – 0.104)	0.015 (0.010 – 0.020)	−0.006 (−0.016 – 0.005)	0.895 (0.833 – 0.957)

Group mean (Hierarchical Error).

While the HBI comparison provided robust evidence that performance was most parsimoniously characterized by a global decay in RT plus the categorical representation of distractor likelihood, it is not possible to tell from this analysis alone whether our models would produce discriminable behavioral patterns given the level of noise present in the data and the best-fit parameters. Specifically, while the accumulator and RL models make subtly different predictions, trial-by-trial random noise in RT could cause both to be most parsimoniously explained by a categorical model, which had one fewer free parameter.

**Figure 4:**
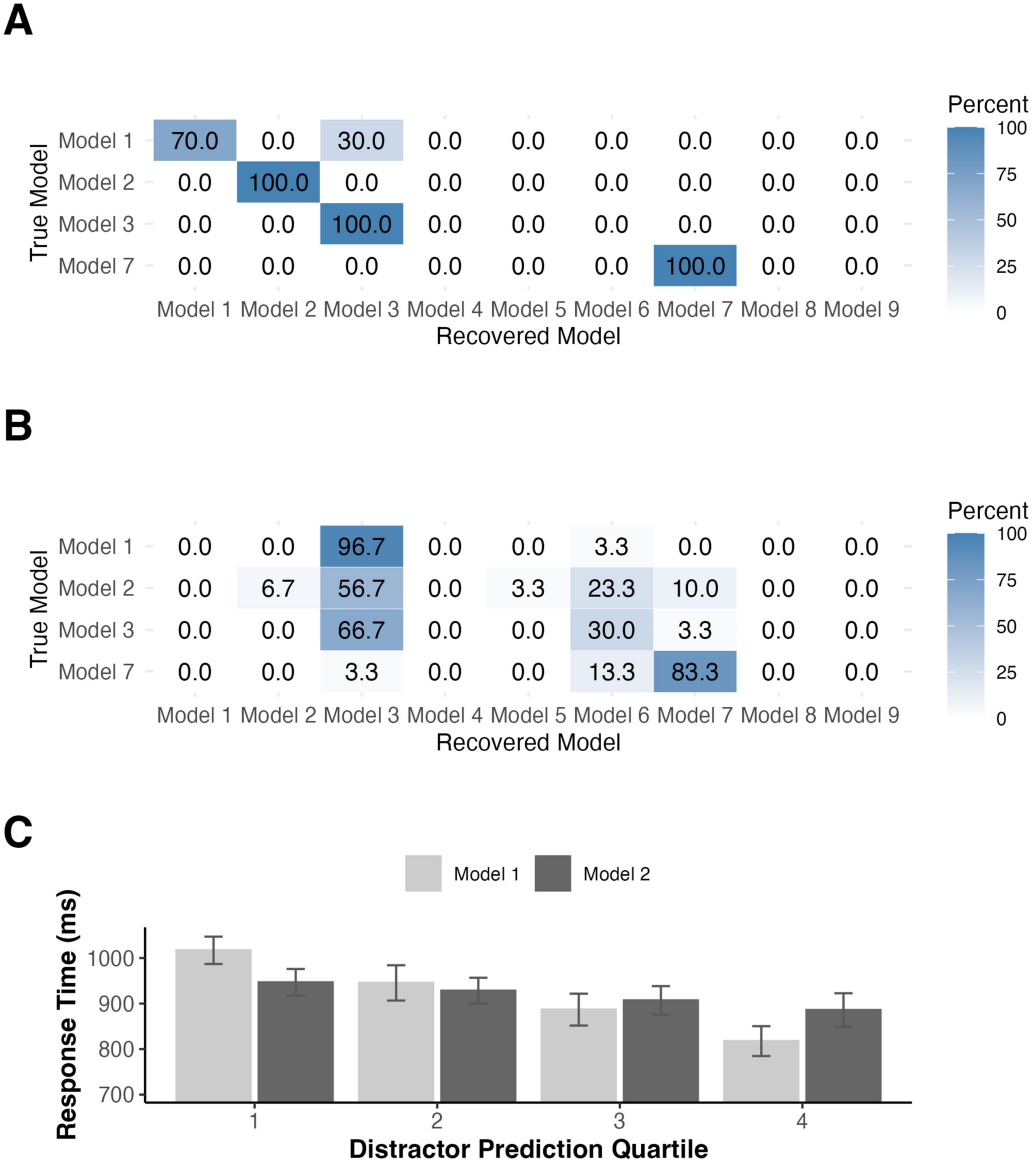
Model recovery simulations for Experiment 1 when noise was based added with **(A)** a standard deviation of 50 ms or **(B)** according to the standard deviation of the residual RT time course in our observed data fits. **(C)** Mean RTs binned according to accumulator (Model 1) or RL (Model 2) derived distractor predictions in Experiment 1. Error bars denote difference- and correlation-adjusted 95% confidence intervals (Cousineau et al., 2021). Correlation adjustment was carried out using the Cousineau-Morey approach (Cousineau, 2005; Morey, 2008).

We first ran a model recovery simulation under conditions of low noise to confirm that HBI was able to detect differences in data generated by the different models under optimal conditions. We simulated data for Models 1, 2, 3, and 7 based on the best-fit parameters for each model fit separately with HBI (see Supplementary Table 1). When only minimal random noise (SD = 50 ms) was added to the simulated data, model recovery was strong with each model except for Model 1 correctly identified on 100% of simulations. Model 1 was misclassified as Model 3 on 30% of simulations suggesting that even under low noise, these models sometimes generated similar patterns of data (see Figure 4A). However, when the noise level in the simulated data was sampled from the standard deviation of each real participant’s residualized RTs, recovery performance declined (see Figure 4B). Specifically, Models 1 and 2 were often misidentified, instead being best fit by Models 3 and 6 on most simulations. Model 6 was identical to Model 3, with the exception that predictions reset at the start of each block, and made highly similar predictions to Model 3. Data generated by Model 3 were correctly identified on 66.67% of simulations with the remaining simulations predominantly best fit by Model 6. Model 7 was misidentified as Model 3 on 3.33% of simulations.

Lastly, for illustration, we binned RTs for the distractor present trials used in model fitting into quartiles according to model-derived distractor predictions based on exponential decline (Model 1) and based on RL (Model 2). In both cases, we observed a decrease in RT as distractor predictions increased, illustrating how accumulator and RL mechanisms converged on a similar explanation of the data (see Figure 4C).

### Discussion

As in previous studies, we found support for location-based distractor suppression. Response times for trials with a singleton distractor appearing at the high probability location were smaller than those for trials where the distractor appeared at a low probability location. We also found no significant differences in high probability distractor trial RTs according to the previous trial type. We fit the data with a series of computational models to determine the nature of the learning mechanism. A binary representation of high and low probability distractor locations paired with a global exponential decline in RT best accounted for the data and we found no significant evidence of trial-by-trial priming. Together, these results suggest there were minimal short-term trial history effects. Instead, a category-like division of trials into high and low probability locations, which necessarily reflects a far-reaching integration of trial history, most parsimoniously explained learned suppression. Of note, while we report *R^2^* as an indicator of how well the best fit model accounted for our data, our goal was to determine which of the relatively simple models included here best accounted for our data. Low to moderate *R^2^* values are therefore not a problem for interpreting our findings, but rather indicate that factors beyond statistical learning of distractor likelihoods may also contribute to moment-by-moment changes in RT.

Our results leave open the possibility that under conditions of reduced noise, differential signatures of accumulator and RL mechanisms could emerge. While model recovery performance was strong when RT variability was low in simulated data, performance declined under conditions of greater variability. Data generated by Models 1 and 2 mimicked Models 3 and 6, suggesting that the differences among accumulator and RL mechanisms were smaller than unexplained variation in trial-by-trial RT. However, it is unlikely that a priming mechanism alone could account for our results since data generated by Model 7 was only rarely misidentified as Model 3.

A limitation of the current study was the inclusion of only 480 trials, only half of which had a distractor present. While this is sufficient for finding robust evidence of suppression, it is possible that an increased number of low probability distractor trials would provide support for a more nuanced probability tracking mechanism. Consequently, in Experiment 2, we replicated these results with more than double the participants per group and over two times as many trials. Furthermore, we tested whether a categorical probability tracking mechanism would still be preferred when individuals regularly experienced changes in distractor probabilities.

## Experiment 2

In Experiment 1, the high probability distractor location remained constant for each participant. While a long temporal integration window and all-or-nothing representation of distractor likelihoods may be advantageous in stable environments with unchanging probabilities, a history of changes in the underlying statistical regularities of the task may promote a gradient of suppression across locations and an increased reliance on short-term trial history. In Experiment 2, we replicated the results in a larger online sample with more than double the number of trials and introduced a between-subjects manipulation of probability stability. For half of the participants, probabilities remained constant just as in Experiment 1. However, for the remaining participants, the high probability distractor location changed every 180 trials such that each location served as the high probability location for each participant over the course of the experiment.

The changing probabilities group allowed us to test whether the distractor likelihood tracking mechanism differed in the face of periodic probability changes. In real world environments, individuals are likely to encounter periodic shifts in distractor regularities, and it is useful to understand whether changes in distractor probabilities are associated with an increased reliance on immediate trial history. Critically, our models made differing predictions about how individuals would respond to changes in distractor likelihood. If participants did not reset distractor expectations and maintained a graded representation of distractor likelihood based on an exponential decay function (Model 1) or suppressed the highest probability distractor location according to an all-or-nothing representation (Model 3), we predicted that participants would continue to suppress previous high probability distractor locations after changes in the statistical structure. This is because under both the accumulator and categorical suppression accounts tested here, estimates of distractor probability do not decrease in the face of conflicting new distractor location information. The exponential model would predict a rise in suppression at the new high probability location while the categorical model would predict that the new high probability location would not be suppressed until the number of distractors having appeared there equaled or surpassed the frequencies for other locations. It is possible that participants would employ a more flexible strategy when faced with periodic changes in the underlying probabilities. In this case, RL would allow participants to decrease the magnitude of suppression at one location while another is increased based on PEs (Model 2). Alternatively, it is possible that participants may reset distractor predictions between blocks when in a more dynamic environment instead of adopting an overall more flexible strategy such as RL. If participants reset distractor probability representations, there would be no carryover effects of previous high probability locations following a change in probabilities, allowing a rapid shift in the representation of distractor likelihood followed by stable maintenance of the new probability structure.

### Materials and methods

#### Participants

The effect size associated with the comparison of RTs across high probability, low probability, and distractor absent trials (*η_p_^2^* = 0.779) from Experiment 1 exceeded that used in our previous power analysis. Given the noisier nature of online data collection, we more than doubled the number of participants in Experiment 1 for each group. A total of 97 individuals (47 female, 1 did not report) ranging in age from 18 to 53 years (*M* = 32.9, *SD* = 6.8) completed the study online and successfully submitted their data through the website Prolific^1^ in exchange for $10 compensation. An additional participant reported being unable to finish because of a technical error. All participants had normal or corrected-to-normal color vision, had a Prolific approval rating between 90 and 100, were currently in the U. S., and had previously completed at least 100 submissions on Prolific. Of the individuals who completed the task, 12 were excluded for having overall behavioral accuracies that were less than 70% and one was excluded for not responding during the final 180 trials of the experiment. For the remaining 84 participants, half received constant distractor location probabilities (counterbalanced across participants so that each location served as the high probability location for seven participants) while the remaining participants periodically experienced a change in underlying distractor location probabilities. All participants read and agreed to a digital consent form that was approved by the Wake Forest University Institutional Review Board. Demographic data were collected through a self-reported Qualtrics survey.

We did not limit the number of times that participants could begin the experiment once accepting it on Prolific to allow them to restart if they experienced technical difficulties. Some participants did restart the study one or more times and received different group assignments and/or high probability distractor locations upon restarting. In most cases, we were unable to tell how many trials such participants completed before restarting. Thus, it is possible that some of the participants completed some trials with a different group assignment before quitting and restarting the task again. Furthermore, participants could have reloaded the task with the same group assignment once it began by refreshing the web page. We elected to not exclude participants based on beginning the task multiple times or refreshing the task page itself since these occurrences were unlikely to occur after the participant had significantly progressed into the task, were likely to be the result of technical difficulties unrelated to the task, and could not account for the effects of interest. Specifically, an inconsistent group assignment would only diminish the location-based suppression effect for participants in the constant probabilities group and would not change the interpretation for participants in the changing probabilities group since these participants still experienced periodic updates in the session for which the data saved.

#### Design and procedure

After completing the demographics questionnaire, participants were redirected to our task, which was hosted on Pavlovia^2^. The task was coded using PsychoJs (version 2023.1.3) as implemented in PsychoPy (Peirce et al., 2019) and participants were required to complete the task using either Firefox or Chrome browsers on a non-mobile device. All aspects of the procedure were the same as Experiment 1 except where noted below. Given the nature of online data collection, we could not control the size of stimuli in terms of visual angle across participants. This did not pose a challenge for our planned analyses since the critical comparison pertained to high and low probability distractor present trials and any differences attributable to stimulus size would equally affect both conditions. Likewise, while online data collection is subject to greater variability in stimulus timing and RT measurement (Bridges et al., 2020), this extra variability would apply equally to all conditions. All participants first completed a task in which they iteratively resized an image of a credit card to match the actual size to compute a scaling factor. This procedure approximately equated the size of the stimuli on each participant’s screen but did not control for viewing distance.

Participants completed a total of 1,080 trials, broken up into 18 blocks of 60 trials each. In between each block participants took a short break and were shown their accuracy. As in Experiment 1, the target appeared at the high probability location on half of all low probability trials to equate the overall number of target appearances at each array location across distractor present trials and the block. However, unlike Experiment 1, the relationship between target position and distractor position for the remainder of low probability trials was randomly assigned and all participants received the same number of target presentations at each location across the experiment. For participants in the changing probabilities group, the high probability distractor location changed every 3 blocks (180 trials) Participants in the constant probabilities had the same high probability distractor location for each block of trials. Participants completed 12 practice trials prior to beginning the task.

#### Data processing

As in Experiment 1, we removed inaccurate trials and trials with RTs less than 200 ms, which accounted for less than 1% of all trials with an accurate response, for the RT analysis. The maximum RT of 2000 ms again served as an upper-bound for all RTs.

### Computational modeling

We again used HBI for model fitting and comparison with the same models as in Experiment 1. Given the possibility that our two groups may use different learning mechanisms, we fit the data for participants in each group separately. Following model fitting and comparison, we again conducted model recovery simulations. For participants in the constant probabilities group, we followed an identical procedure as in Experiment 1. We followed the same procedure for participants in the changing probabilities group, except when selecting the data generating models. To best account for the changing probabilities and in light of the model comparison results, we generated data from the set of models that included a reset at the start of each block (Models 4–6) in addition to our priming and global decay model (Model 7).

### Results

**Figure 5:**
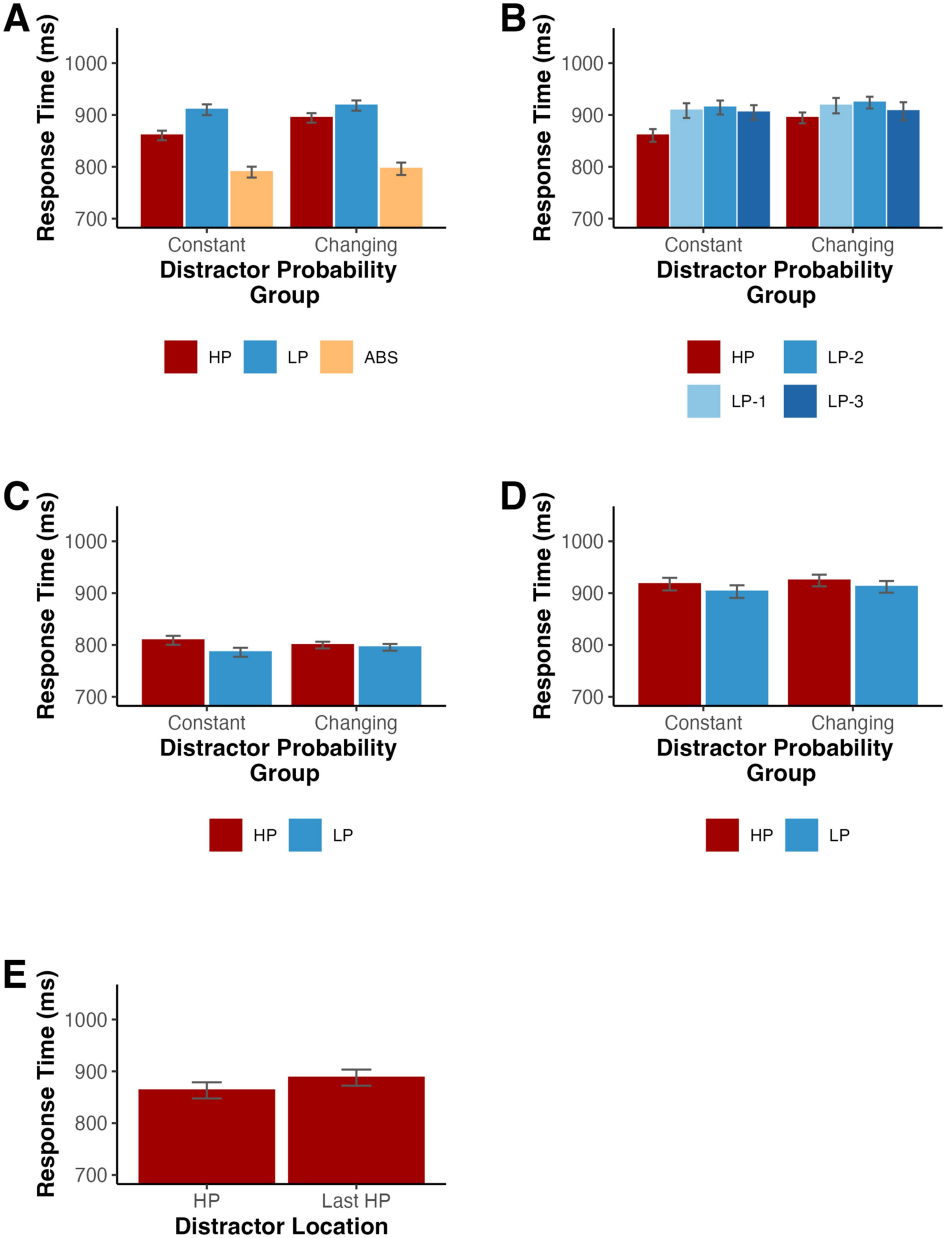
**(A,B)** Mean RTs depending on the distractor location for Experiment 2. **(C)** Mean RTs for distractor absent trials depending on the target location for Experiment 2. **(D)** Mean RTs for low probability distractor trials depending on the target location for Experiment 2. HP loc indicates the high-probability location, LP-1 indicates one item away from the high-probability location, LP-2 indicates two items away, LP-3 indicates three items away, and ABS indicates when the distractor was absent. **(E)** Mean RTs for participants in the changing probabilities condition according to whether the distractor was a current high probability location (HP) or the most recent high probability location (Last HP). Note the mean for HP trials is different than in **(A,B)** because the first three blocks are excluded from analysis. Error bars denote difference- and correlation-adjusted 95% confidence intervals (Cousineau et al., 2021). Correlation adjustment was carried out using the Cousineau-Morey approach (Cousineau, 2005; Morey, 2008).

A mixed ANOVA on mean RTs with distractor location (high-probability, low-probability, and distractor absent) and probability group (constant and changing) as factors indicated a significant difference existed among distractor locations, *F*(2,164) = 601.81, *p* < 0.001, *η_p_^2^* = 0.880, as well as a significant interaction of location by group, *F*(2,164) = 9.24, *p* < 0.001, *η_p_^2^* = 0.101. Since the interaction was significant, we repeated the ANOVA for each group separately. There was a significant difference in RT among the distractor locations for participants in both the constant probabilities, *F*(2,82) = 296.31, *p* < 0.001, *η_p_^2^* = 0.878, and changing probabilities, *F*(2,82) = 314.08, *p* < 0.001, *η_p_^2^* = 0.885, groups (see Figure 5A). There was no significant main effect of probability group, *F*(1, 82) = 0.35, *p* = 0.554, *η_p_^2^* = 0.004. Furthermore, we tested distractor present trials only in an additional repeated measures ANOVA and found that the difference in RT between low probability and high probability distractor trials was greater for participants in the constant probabilities group than in the changing probabilities group, *F*(1,82) = 16.48, *p* < 0.001, *η_p_^2^* = 0.167.

We conducted follow-up Holm-Bonferroni-corrected post-hoc comparisons separately for individuals in the constant version and individuals in the changing version of the task. For the constant version, RTs were longer when the distractor was present in the low-probability locations, *t*(41) = 22.53, *p* < 0.001, *d_z_* = 3.48, and when the distractor was present in the high-probability location, *t*(41) = 14.70, *p* < 0.001, *d_z_* = 2.27, relative to distractor absent trials. Importantly, RTs were again shorter when the distractor appeared in the high-probability location compared to the low-probability locations, *t*(41) = 10.48, *p* < 0.001, *d_z_* = 1.62. Participants in the changing probabilities group showed an identical pattern. RTs were longer when the distractor was present in the low-probability locations, *t*(41) = 21.38, *p* < 0.001, *d_z_* = 3.30, and when the distractor was present in the high-probability location, *t*(41) = 18.16, *p* < 0.001, *d_z_* = 2.80, relative to distractor absent trials. Again, RTs were shorter when the distractor appeared in the high-probability location compared to the low-probability locations, *t*(41) = 5.60, *p* < 0.001, *d_z_* = 0.86.

As in Experiment 1, we examined the spatial gradient of suppression and split the low-probability trials into 3 conditions based on the distance from the high-probability location. When testing the effect of distractor location (high-probability, 1 item away, 2 items away, and 3 items away) and probability group (constant and changing) on RTs with a mixed ANOVA, we found a significant main effect of distractor location, *F*(3,246) = 29.86, *p* < 0.001 (Geisser–Greenhouse corrected for violation of the sphericity assumption), *η_p_^2^* = 0.267, and a distractor location by group interaction, *F*(3,246) = 4.12, *p* = 0.010 (Geisser–Greenhouse corrected for violation of the sphericity assumption), *η_p_^2^* = 0.048 (see Figure 5B). The main effect of probability group failed to reach statistical significance, *F*(1,82) = 0.26, *p* = 0.614, *η_p_^2^* = 0.003. We calculated the slope of the mean RTs for the three low probability locations for each participant. A series of one-sample *t*-tests revealed that these slopes did not differ from 0 for both the constant probabilities (*M* = −1.85, *SD* = 23.10), *t*(41) = −0.52, *p* = 0.606, *d_z_* = −0.08, and changing probabilities (*M* = −5.33, *SD* = 28.99), *t*(41) = −1.19, *p* = 0.241, *d_z_* = −0.18, groups.

Next, we tested whether RTs on distractor absent and low probability distractor trials varied according to the location of the target with a mixed ANOVA on mean RTs with target location (high-probability and low-probability) and probability group (constant and changing) as factors. For distractor absent trials, there was a significant main effect of target location, *F*(1,82) = 26.15, *p* < 0.001, *η_p_^2^* = 0.242, and a significant interaction of location by group, *F*(1,82) = 12.14, *p* < 0.001, *η_p_^2^* = 0.129, such that the difference in RT between trials with high and low probability location targets was larger for participants in the constant probabilities group than for those in the changing probabilities group (see Figure 5C). The main effect of probability group again failed to reach significance, *F*(1,82) < 0.01, *p* = 0.996, *η_p_^2^* < 0.001. Follow-up t-tests revealed that participants in the constant probabilities group, *t*(41) = 5.38, *p* < 0.001, *d_z_* = 0.83, but not those in the changing probabilities group, *t*(41) = 1.36, *p* = 0.183, *d_z_* = 0.21, were significantly slower when the target appeared in the high probability location. When testing low probability distractor trials, participants were slower when the target appeared at the high probability distractor location than when it appeared at a low probability distractor location, *F*(1,82) = 10.52, *p* = 0.002, *η_p_^2^* = 0.114 (see Figure 5D). Neither the main effect of probability group, *F*(1,82) = 0.09, *p* = 0.768, *η_p_^2^* = 0.001, nor the interaction of target location by probability group, *F*(1,82) = 0.07, *p* = 0.792, *η_p_^2^* < 0.001, reached statistical significance.

The changing probabilities condition in Experiment 2 allowed us to test whether RTs for previous high probability locations continued to receive suppression even after a change in the underlying probabilities. We excluded trials where the distractor appeared at a location that had not yet been associated with a high probability or that appeared at a location associated with a high probability prior to the most recently preceding context. The number of trials in these potential conditions varied over the course of the experiment and could be biased by an overall trend such as that observed in Experiment 1. Likewise, we excluded the first three blocks from the analysis since there was no previous high probability location prior to the fourth block. Response times were significantly shorter for current high probability location trials relative to previous high probability location trials, indicating ongoing adjustments in priority in response to the probability changes, *t*(41) = 3.19, *p* = 0.003, *d_z_* = 0.49 (see Figure 5E).

We analyzed behavioral accuracies with a mixed ANOVA with distractor location (high-probability location, low-probability location, and absent) and probability group (constant and changing) as factors. There was a significant difference among distractor locations, *F*(2,164) = 61.48, *p* < 0.001 (Geisser–Greenhouse corrected for violation of the sphericity assumption), *η_p_^2^* = 0.428. However, there was no difference in accuracy between the constant and changing probability groups, *F*(1,82) = 0.28, *p* = 0.597, *η_p_^2^* = 0.003, and there was no significant interaction, *F*(2,164) = 0.55, *p* = 0.523 (Geisser–Greenhouse corrected for violation of the sphericity assumption), *η_p_^2^* = 0.007 (see Table 3).

**Table 3:** Behavioral accuracies for Experiment 2.

	High probability distractor	Low probability distractor	Distractor absent
Constant probabilities	95.42 (4.06)	93.57 (6.25)	97.01 (2.96)
Changing probabilities	94.55 (4.84)	93.10 (5.88)	96.81 (3.55)

Mean accuracy (SD).

#### Mechanisms of learning

**Figure 6:**
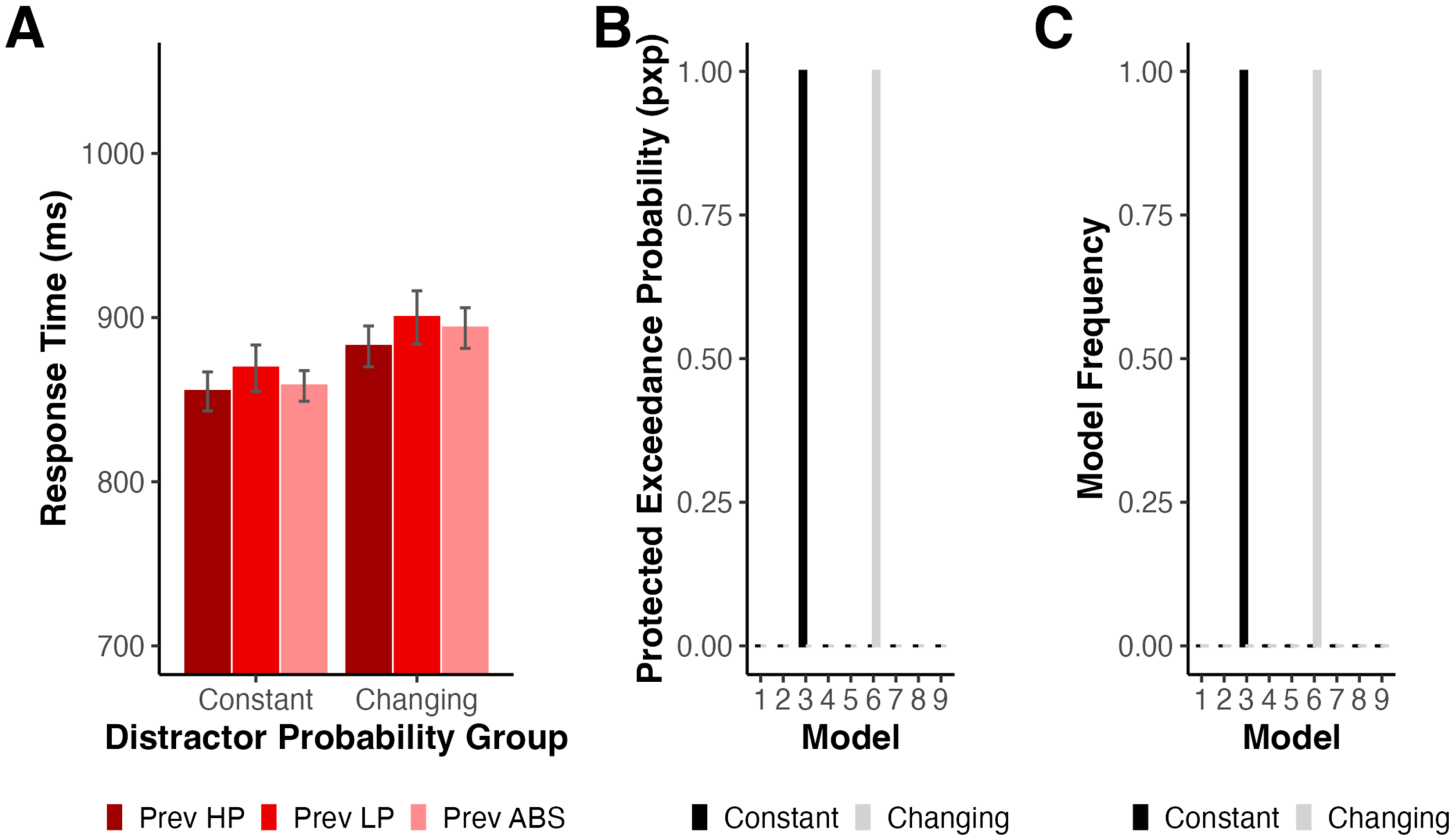
**(A)** Mean RTs for high probability distractor trials depending on the previous trial type and probability group for Experiment 2. Error bars denote difference- and correlation-adjusted 95% confidence intervals (Cousineau et al., 2021). Correlation adjustment was carried out using the Cousineau-Morey approach (Cousineau, 2005; Morey, 2008). **(B)** Protected Exceedance Probability for model comparison in Experiment 2. **(C)** Proportion of participants whose data were best explained by each model in Experiment 2.

As in Experiment 1, we first examined the impact of immediate trial history on subsequent RTs by testing whether RTs on high probability distractor trials varied as a function of the preceding trial type, excluding the first trial of each block and trials where the target location repeated. We conducted a mixed ANOVA with factors of preceding trial type (high probability, low probability, and distractor absent) and probability group (constant and changing). There was a significant main effect of preceding trial type, *F*(2,164) = 6.22, *p* = 0.004 (Geisser–Greenhouse corrected for violation of the sphericity assumption), *η_p_^2^* = 0.070, but no significant main effect of probability group, *F*(1,82) = 1.20, *p* = 0.277, *η_p_^2^* = 0.014, or significant interaction *F*(2,164) = 0.38, *p* = 0.656 (Geisser–Greenhouse corrected for violation of the sphericity assumption), *η_p_^2^* = 0.005 (see Figure 6A). We conducted a series of post-hoc Bonferroni-Holm-corrected pairwise comparisons collapsed across probability groups. Participants had shorter RTs following high probability distractor location trials relative to low probability distractor location trials, *t*(83) = 3.13, *p* = 0.007, *d_z_* = 0.34, and a similar trend existed when compared against distractor absent trials, *t*(83) = 2.05, *p* = 0.086, *d_z_* = 0.22. There was also a trend such that RTs were larger following low probability trials than distractor absent trials, *t*(83) = 1.83, *p* = 0.086, *d_z_* = 0.20.

Given that high probability location repetitions were associated with a reduction in RT, we repeated the comparison of high probability, low probability and distractor absent RTs with trial-by-trial distractor and target repetitions excluded. For this analysis, the first trial of each block was again excluded. As in the main analysis, there was a significant main effect of distractor condition, *F*(2,164) = 503.12, *p* < 0.001 (Geisser–Greenhouse corrected for violation of the sphericity assumption), *η_p_^2^* = 0.860 as well as a significant interaction of probability group by distractor condition, *F*(2,164) = 8.70, *p* < 0.001 (Geisser–Greenhouse corrected for violation of the sphericity assumption), *η_p_^2^* = 0.096. The difference in RT between high probability and low probability distractor trials remained significant for both conditions, *t*s > 4.12, *p*s < 0.001.

We again fit and compared a series of computational models to test the mechanisms responsible for learned location-based distractor suppression. While the constant group served as a replication of Experiment 1, the changing group allowed us to ask whether participants would represent distractor probabilities differently when each location served as the high probability location across the entire experiment. For participants in the constant probability group, the winning model again accounted for distractor present RTs according to a global exponential decline paired with categorical suppression of the location with the highest distractor frequency count (Model 3), pxp = 1.00, bor < 0.001 (see Figures 6B,C). As in Experiment 1, we computed *R^2^* for each participant by taking the best-fit parameters from HBI and rerunning the model for each participant individually. *R^2^* ranged from 0.01 to 0.54 (*M* = 0.17, *SD* = 0.11). The best-fit group parameters are displayed in Table 4. We tested whether each GLM parameter estimate differed from 0 with a series of HBI *t*-tests. Participants had shorter RTs when the distractor appeared at the location with the highest summed distractor frequency, *t*_*HBI*_(43.00) = −8.77, *p* < 0.001, and demonstrated significant global exponential decline in RT, *t*_*HBI*_(43.00) = 11.90, *p* < 0.001, but did not differ based on whether the previous trial had a distractor present, *t*_*HBI*_(43.00) = 0.31, *p* = 0.758.

**Table 4:** Constant probability group parameters for Model 3 in Experiment 2.

Distractor prediction parameter estimate	Global decay parameter estimate	Decay rate	Previous trial distractor absent parameter estimate	Intercept
−0.048 (−0.054 – −0.043)	0.077 (0.071 – 0.084)	0.010 (0.009 – 0.012)	0.001 (−0.003 – 0.006)	0.887 (0.858 – 0.916)

Group mean (Hierarchical Error).

We next repeated the same procedure for the changing probabilities group. When distractor probabilities periodically changed, participants’ data were again best explained by a categorical model, but the winning model reset predictions between blocks (Model 6), pxp = 1.00, bor < 0.001. The degree to which the winning model accounted for individual subjects’ data ranged from 0.02 to 0.51 (*M* = 0.16, *SD* = 0.12). Participants had shorter RTs on trials where the distractor appeared at the most frequent location (reset with each block), *t*_*HBI*_(43.00) = −4.22, *p* < 0.001, and there was a significant global exponential decline in RT, *t*_*HBI*_(43.00) = 10.94, *p* < 0.001 (see Table 5). RTs did not differ between previous distractor absent and previous distractor present trials, *t*_*HBI*_(43.00) = 0.71, *p* = 0.483.

**Table 5:** Changing probability group parameters for Model 6 in Experiment 2.

Distractor prediction parameter estimate	Global decay parameter estimate	Decay rate	Previous trial distractor absent parameter estimate	Intercept
−0.022 (−0.027 – −0.017)	0.083 (0.075 – 0.090)	0.010 (0.008 – 0.011)	0.003 (-0.001 – 0.008)	0.892 (0.864 – 0.920)

Group mean (Hierarchical Bayesian Error).

**Figure 7:**
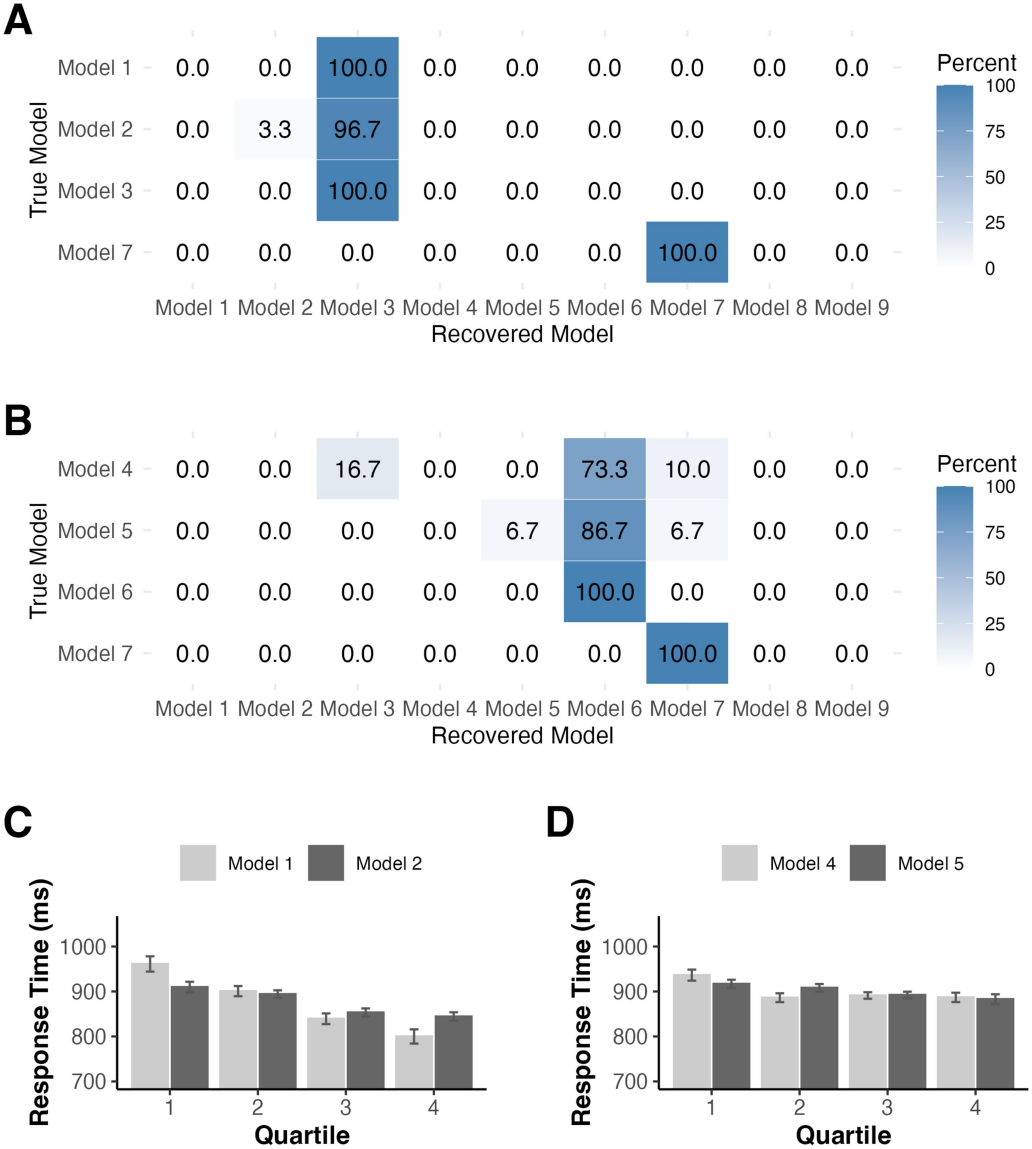
Model recovery simulations for Experiment 2 for participants in the **(A)** constant probabilities group and for those in the **(B)** changing probabilities group. Noise standard deviations were derived from fitting our observe data. **(C)** Mean RTs binned according to accumulator (Model 1) or RL (Model 2) derived distractor predictions for constant probability participants in Experiment 2. **(D)** Mean RTs binned according to accumulator (Model 4) or RL (Model 5) derived distractor predictions for changing probability participants in Experiment 2. Error bars denote difference- and correlation-adjusted 95% confidence intervals (Cousineau et al., 2021). Correlation adjustment was carried out using the Cousineau-Morey approach (Cousineau, 2005; Morey, 2008).

As in Experiment 1, we followed up the HBI model comparison with a series of model recovery simulations. Since model recovery was strong when noise was low in Experiment 1, we focus here only on noise levels estimated from the residuals after fitting our observed data with each model (see Supplementary Tables 2, 3 for best-fit parameters) The results of our simulations for the constant probabilities condition were similar to those from Experiment 1 (see Figure 7A). Models 1 and 2 were frequently misidentified as Model 3 and data generated by Models 3 and 7 were correctly identified on 100% of simulations. Given that a prediction resetting model won the comparison for the changing probabilities group, we generated data from Models 4–7 to simulate model recovery in non-stationary environments using the trial sequences from the changing probabilities condition. Models 4 and 5 were misidentified as Model 6 on nearly all simulations (see Figure 7B). Models 6 and 7 were correctly identified on 100% of simulations. For illustration, we again binned the RTs used in model fitting for participants in both groups according to model-derived distractor predictions based on Models 1 and 2 for the constant probabilities group and Models 4 and 5 for the changing probabilities group (see Figures 7C,D).

### Discussion

As in Experiment 1, we found evidence of learned distractor suppression. When examining the RT data, we found significant interactions between the probability group and the distractor location such that while participants in both groups demonstrated suppression of the high probability location, the magnitude of suppression was stronger for participants who received constant probabilities. Most interestingly, the data from both groups were again best explained by a category-like suppression of the most likely distractor location. However, participants in the constant probabilities group integrated distractor predictions across blocks while those in the changing probabilities group reset distractor estimates to account for the probability changes.

The model recovery simulations were consistent with those from Experiment 1. Data simulated in the constant probabilities group according to the best-fit parameters from Models 1–3 were almost always identified as Model 3, indicating that accumulator and RL mechanisms mimicked categorical suppression. Importantly, Model 7 was never misidentified as Model 3, indicating that these misclassifications were specific to the models with a learning mechanism. Likewise, data generated according to Models 4–6 in the changing probabilities condition were almost always identified as Model 6.

Unlike Experiment 1, we did find a significant relationship between immediate trial history and performance. Participants demonstrated less capture on high probability distractor trials that followed other high probability trials than those following low probability trials, and this effect did not significantly differ between groups. Together, our results suggest that a combination of short-term priming and longer-term frequency tracking best account for learned location-based distractor suppression.

## General discussion

In the current study, we investigated how individuals harness statistical learning to guide the allocation of attention. In replication of a growing body of research, we found that participants learned the location a salient distractor tended to appear with higher frequency and suppressed attentional selection of this location. Most importantly, using HBI, we compared a series of computational models that differed in whether individuals track distractor probabilities by summing evidence across trials, nudging distractor location predictions up and down according to an RL mechanism, or adopting a stable category-like representation of high and low probability locations. Across two experiments, participants’ RT data were most parsimoniously explained by a combination of a global exponential decline and a category-like suppression of the most highly associated distractor location. Participants who received constant probability structures appeared to base this categorization on evidence spanning experimental blocks and those who received changing probability structures appeared to periodically reset their probability estimations.

Our results suggest that after accounting for a global exponential decline in RT that occurs with each distractor presentation, the magnitude of suppression afforded spatial locations remains stable and resistant to change. This finding is consistent with a long temporal integration window in which individuals rely on extended trial histories and do not make strong adjustments in predictions based on rare, unexpected distractor appearances. Importantly, our findings do not provide evidence that is necessarily contrary to the existence of either accumulator or RL mechanisms since categorical suppression must still rely on some form of probability tracking and both serve as potential candidates. For simplicity, our categorical model relied on frequency accumulation, meaning that after several high probability location trials, this location would always receive preferential treatment as the most highly distractor associated location. However, our design does not allow us to rule out other probability tracking mechanisms that could be paired with the categorization of high and low probability locations. Our data suggest that the strength of suppression, at least as it is detectable in the current task, may relate more strongly to the coarse differences in probabilities across locations (see Lin et al., 2021) than to fine-grained differences in the number of times a distractor has appeared at any particular location. While our data do not conclusively point to a single learning mechanism, they do suggest that individuals weigh long-term trends over recent evidence.

Under conditions of low noise, model recovery simulations largely discriminated between accumulator, RL, and categorical model-generated data, suggesting that with low enough noise, these mechanisms should be separable, even when the temporal integration window is long. Here, we did not explicitly manipulate trial history other than by setting the overall distractor probabilities per block. Future studies with more targeted manipulations of local trial history (in addition to the global manipulation used here) may be better equipped to differentiate between low decay accumulator and low learning rate RL models. Nonetheless, our data suggest that simple categorization of high and low probability locations accounts for performance at least as well as more complicated models that adjust predictions both up and down in a continuous fashion.

The introduction of a condition in which probabilities periodically changed in Experiment 2 was meant to parallel real-world scenarios in which statistical regularities are not always constant. We observed evidence in favor of category-like suppression even under these non-stable conditions. Our results suggest that individuals make rapid reconfigurations of distractor location expectations in the face of probability changes that are then stably maintained and have implications for how learned suppression may function outside of laboratory tasks. While we also observed significant trial-by-trial priming of location for high probability distractor trials in Experiment 2, this priming effect did not differ across our two groups. Importantly, we still found evidence of learned distractor suppression when excluding trials with distractor and target location repetitions.

To equate the number of target presentations at each location across all trials, the target appeared at the high probability location on half of all low probability distractor trials. It is possible that this relationship contributed to the adoption of a categorical representation of distractor locations. For example, the target position was less variable on low distractor probability than on high distractor probability trials and this shared property could lead participants to chunk all low probability locations together rather than updating representations individually. A reliance on the distractor location to target relationship on low probability trials would facilitate search by guiding participants to the expected target location (e.g., Chun and Jiang, 1998). Therefore, such an effect should only minimize the difference in RT between high and low probability distractor trials. Instead, we observed robust evidence of location-based suppression that varied according to distractor position frequencies with participants demonstrating reduced RTs on trials where the distractor appeared at the categorized high probability location.

While previous studies have not explicitly tested how individuals update distractor location predictions, our results are consistent with findings that statistical learning for distractor suppression is in some cases relatively inflexible. Distractor suppression persists into extinction in the absence of new competing probabilities (Britton and Anderson, 2020). Furthermore, when a basic feature such as color serves as a context cue for which of two locations has a high likelihood of containing a salient distractor, individuals demonstrate suppression of both locations, regardless of changes in cue, again reflecting a carryover of previous learning (de Waard et al., 2022). The categorical hypothesis that our data support is consistent with both patterns since under this model suppression is shielded from trial-by-trial variability in outcomes. Since we only changed probabilities between blocks, participants may have represented each block as a potentially new context, allowing participants in the changing probabilities group to form new rule-like representations at block transitions. This would be in line with other evidence that learned suppression can be context-specific given strong cues (de Waard et al., 2023). Although de Waard et al. (2023) demonstrated that participants reinstated suppression for all previous high probability locations, we did not observe a carryover of previous distractor position learning. This difference may be due to the always biased nature of distractor probabilities in our design. Finally, we did not collect data on participants’ awareness of the probability manipulations, so it is possible that some participants were explicitly aware of the statistical structure. However, similar studies have found that learned suppression often operates outside of explicit awareness (Ferrante et al., 2018, 2023; Wang and Theeuwes, 2018b).

It is also important to note that the use of learning mechanisms may themselves be context dependent. For example, individuals may sometimes employ one learning mechanism but flexibly switch mechanisms in response to expectations about the stability of the statistical structure of the world around them. For example, it is possible that participants in the current study quickly adopted a binary representation of high and low probability locations because all low probability locations received an equal number of distractors within each block and were thus always roughly equated in trial history. To differentiate between these possibilities, future designs may use more nuanced probability structures, such as varying the distractor likelihoods among low probability locations. Relatedly, when in an environment in which the high probability location sometimes changes, a shorter temporal integration window would allow individuals to flexibly update expectations and respond to new demands. While our changing probabilities group did experience periodic updates in the underlying statistical structure of the task, these changes still only occurred every 180 trials. An important topic for future research is to test whether reliance on short-term history increases in environments with less stability than tested here.

Our results suggest that statistical learning influences distractor suppression through a category-like representation of high and low distractor likelihood locations. These rule-like representations of high and low probability locations are most strongly based on the overall difference in probabilities across locations rather than trial-by-trial history and allow for stable location-based suppression.

## Data Availability

The datasets presented in this study can be found in the Open Science Framework online repository: https://doi.org/10.17605/OSF.IO/J7BN2.

## Footnotes

^1^ https://www.prolific.com

^2^ https://pavlovia.org

## Author’s note

Experiment 1 of the current study first appeared in a Master’s thesis (Seitz, 2023) and as a conference abstract (Sali and Seitz, 2024).
