# Supplementary material for: Category-like representation of statistical regularities allows for stable distractor suppression
Source: Front Psychol. 2025 Aug 6;16:1598594. doi: 10.3389/fpsyg.2025.1598594 (PMC12365932; doi:10.3389/fpsyg.2025.1598594)
Supplement: Supplementary file 2 [file Table_2.docx]

**Supplemental Table 2.** Group parameters and residual RT time course standard deviations used for model recovery simulations for continuous probabilities in Experiment 2

|  | Distractor Prediction Parameter Estimate | Global Decay Parameter Estimate | Global Decay Rate | Location Decay Rate / Learning Rate | Distractor Repetition Parameter Estimate | Previous Trial Distractor Absent Parameter Estimate | Intercept | Standard Deviation of Residual RTs |
| --- | --- | --- | --- | --- | --- | --- | --- | --- |
| M1 | -0.030 ( -0.034 –  -0.026) | 0.060 (0.053 – 0.066) | 0.009 (0.008 – 0.011) | 0.021 (0.018 – 0.025) | – | 0.001 ( -0.003 – 0.006) | 0.854 (0.826 –0.883) | 0.195 (0.043) |
| M2 | -0.025 ( -0.029 –  -0.021) | 1.079 (1.072 – 1.086) | 0.010 (0.008 – 0.012) | 0.140 (0.121 – 0.162) | – | 0.002 ( -0.003 – 0.006) | 0.854 (0.825 –0.883) | 0.195 (0.043) |
| M3 | -0.048 ( -0.054 –  -0.043) | 0.077 (0.071 – 0.084) | 0.010 (0.009 – 0.012) | – | – | 0.001 ( -0.003 – 0.006) | 0.887 (0.858 –0.916) | 0.195 (0.043) |
| M7 | – | 0.077 (0.071 – 0.084) | 0.011 (0.009 – 0.012) | – | -0.031 ( -0.037 – -0.025) | -0.012 ( -0.017 – -0.008) | 0.868 (0.839 –0.897) | 0.197 (0.042) |

*Note.* We report group means for each parameter with hierarchical Bayesian errors in parentheses. For the standard deviation of residual RTs, we report group means with standard deviation in parentheses. Each model was fit with HBI in isolation and may therefore differ from the best-fit parameters derived from the full model comparison.
